# The Consequences of Feminization in Breeding Groups of Wild Fish

**DOI:** 10.1289/ehp.1002555

**Published:** 2010-10-08

**Authors:** Catherine A. Harris, Patrick B. Hamilton, Tamsin J. Runnalls, Veronica Vinciotti, Alan Henshaw, Dave Hodgson, Tobias S. Coe, Susan Jobling, Charles R. Tyler, John P. Sumpter

**Affiliations:** 1 Institute for the Environment, Brunel University, Uxbridge, United Kingdom; 2 School of Biosciences, University of Exeter, Exeter, United Kingdom; 3 School of Information Systems, Computing and Mathematics, Brunel University, Uxbridge, United Kingdom; 4 Environment Agency National Coarse Fish Farm, Calverton, United Kingdom

**Keywords:** DNA microsatellites, ecotoxicology, genetic diversity, intersex, reproductive success, roach, secondary sexual characteristics, size, sperm viability

## Abstract

**Background:**

The feminization of nature by endocrine-disrupting chemicals (EDCs) is a key environmental issue affecting both terrestrial and aquatic wildlife. A crucial and as yet unanswered question is whether EDCs have adverse impacts on the sustainability of wildlife populations. There is widespread concern that intersex fish are reproductively compromised, with potential population-level consequences. However, to date, only *in vitro* sperm quality data are available in support of this hypothesis.

**Objective:**

The aim of this study was to examine whether wild endocrine-disrupted fish can compete successfully in a realistic breeding scenario.

**Methods:**

In two competitive breeding experiments using wild roach (*Rutilus rutilus*), we used DNA microsatellites to assign parentage and thus determine reproductive success of the adults.

**Results:**

In both studies, the majority of intersex fish were able to breed, albeit with varying degrees of success. In the first study, where most intersex fish were only mildly feminized, body length was the only factor correlated with reproductive success. In the second study, which included a higher number of more severely intersex fish, reproductive performance was negatively correlated with severity of intersex. The intersex condition reduced reproductive performance by up to 76% for the most feminized individuals in this study, demonstrating a significant adverse effect of intersex on reproductive performance.

**Conclusion:**

Feminization of male fish is likely to be an important determinant of reproductive performance in rivers where there is a high prevalence of moderately to severely feminized males.

There is international concern regarding the effects of natural and man-made chemicals on the health of humans and wildlife. Estrogenic and antiandrogenic chemicals [so-called endocrine-disrupting chemicals (EDCs)] are of particular concern to aquatic ecosystems, because these compounds are present in almost all treated sewage effluents and in lowland rivers receiving these effluents throughout Europe, Asia, and the United States (Desbrow et al. 1998; Hinck et al. 2009; Hotchkiss et al. 2008; Sumpter and Johnson 2008; Sun et al. 2008). Steroid estrogens in particular can be biologically active at very low concentrations (in the low nanogram per liter range) and are known to cause altered sex ratios (Lange et al. 2008; Länge et al. 2001) and feminization of male fish (Hahlbeck et al. 2004). Feminized phenotypes include the presence of vitellogenin in the blood of male fish (Purdom et al. 1994) and the presence of developing eggs (oocytes) and/or female reproductive ducts (oviducts) in the testes of otherwise male fish (the intersex condition) (Jobling et al. 1998). Although these conditions have been widely reported in both freshwater and marine fish species (Allen et al. 1999; Bjerregaard et al. 2006; Blazer et al. 2007; De Metrio et al. 2003; Hinck et al. 2009; Jobling et al. 1998; Penaz et al. 2005; Stentiford and Feist 2005), there is no evidence that their existence directly affects numbers in wild fish populations.

Some of the most compelling evidence for intersex in wild fish comes from studies on roach (*Rutilus rutilus*) inhabiting U.K. rivers contaminated with sewage effluents. Intersex roach (male fish with developing eggs in their testes) have been found at 86% (of a total of 51) of U.K. river locations (Jobling et al. 2006). Severely intersex fish produce less milt (semen), the milt has a lower sperm density, and the sperm have reduced motility, compared with apparently normal male fish from less contaminated sites (Jobling et al. 2002). Fertilization success—as measured through *in vitro* studies that have determined the proportion of eggs successfully fertilized by intersex fish and the number of these fertilized eggs capable of giving rise to live offspring—is also reduced with increasing severity of the intersex condition (Jobling et al. 2002).

Data such as those described above naturally lead to concern that EDCs may have detrimental effects on fish populations. A limited number of studies have been undertaken to investigate this possibility. Long-term studies in an experimental lake in northwestern Ontario, Canada, showed that exposure to the potent estrogen ethinylestradiol, at 4–6 ng/L over a period of 3 years resulted in the collapse of the population of the fathead minnow (*Pimephales promelas*) (Kidd et al. 2007), but no adverse effects have yet been found on populations of the longer-lived fish species pearl dace (*Margariscus margarita*) and lake trout (*Salvelinus namaycush*) (Palace et al. 2006; Werner et al. 2006). Modeling approaches, using information from experimental exposures, have predicted that concentrations of EDCs found in the environment could lead to population declines, mainly resulting from reduced female, rather than male, fecundity (Grist et al. 2003; Gutjahr-Gobell et al. 2006; Miller and Ankley 2004). Recently, another modeling study, using data obtained from wild fish (Jobling et al. 2002), predicted intersex on the whole to have a minimal effect on the population growth rate in roach but suggested that it may increase the risk to local roach populations when present in combination with selective fishing practices (An et al. 2009).

A critical gap in the knowledge and information that have fed population models published to date is how successfully intersex fish reproduce when competing with apparently normal males in breeding populations. In the wild, roach compete in groups to fertilize the eggs of spawning females (Diamond 1985; Wedekind 1996), so it is important to know whether intersex fish might be at a disadvantage in these competitive spawning situations. In the present study, we attempted to fill this knowledge gap by allowing groups of roach—representative of a naturally spawning shoal containing both normal and intersex fish—to spawn naturally in large tanks and using DNA microsatellites to assign the offspring to the parents. In this way, we assessed the abilities of intersex fish to compete with one another (and with other male fish) and to contribute to the next generation.

## Materials and Methods

### Experimental design

We conducted two experiments, the first in 2006 (study 1) and the second in 2008 (study 2). These studies were designed specifically to examine the effect of long-term exposure of wild male fish to endocrine disruptors. The effects of these chemicals on male gonadal histology are well known, whereas there is currently little evidence that long-term EDC exposure affects female histology in the wild. For each of the two experiments, we collected adult roach using standard electro-fishing methods from wild populations living in effluent-contaminated rivers in the United Kingdom. The sites were chosen based on data from previous national surveys that indicated where roach with varying degrees of intersex were likely to occur (Jobling et al. 2006).

We collected fish in late April, shortly before the natural spawning season, in cooperation with the U.K. Environment Agency. We selected the larger adult fish from those that were caught in order to avoid the inclusion of sexually immature fish and to maximize the chances of including more severely intersex individuals in the study because prevalence and severity of the intersex condition increase with age (Jobling et al. 2006). We then transported the fish to a holding facility (the Environment Agency’s fish breeding unit at Calverton), where we separated them into groups of males and females, based on their body morphotype and the presence of secondary sex characteristics (Kortet et al. 2003). We placed the fish into large fiberglass tanks (each containing kakabans, aquatic weed-like spawning substrates) receiving water via a recirculating system, which was topped up with borehole water, as necessary. Water temperatures were initially set to match ambient temperatures in the river where the fish were collected and were subsequently increased to 15°C over 2 days to encourage ovulation of the females.

The experimental design involved placing six males with three females to create competition between the males (and intersex fish) for the females. We first allocated males to the spawning tanks and allowed them to acclimatize. Spermiation in male roach can occur in captivity, and males can produce sperm over a period of a few weeks without the need for any intervention. For females, synchronicity of spawning was required to enable removal of the adults as soon as possible after spawning (to minimize fish eating the spawned eggs). This was achieved by injection of females with carp pituitary extract at a time just prior to natural ovulation. We then placed three females into each tank with the males to allow spawning. Subsequently, some tanks were found to contain five males and four females, because the external features used for classification of sex can be affected by exposure to EDCs contained in effluent discharges.

All animals used in this research were treated humanely and with regard for the alleviation of suffering; all procedures were subject to approval by the local ethical review process as required under the U.K. Animals (Scientific Procedures) Act (1986).

### Study 1: 2006 breeding experiment

We collected fish on 24 April 2006 from a 200-m stretch immediately downstream of Chertsey sewage treatment works on the River Bourne (Surrey, UK; 51°24′08″N; 0°32′07″W). Seven breeding tanks were used, with a total of 63 fish, including 38 males (Table 1).

### Study 2: 2008 breeding experiment

The 2008 experiment included a greater number of fish—and more severely intersex fish—than were included in 2006, and was designed to reduce the confounding effect of size on reproductive success. Fish were obtained from the River Arun (Sussex, UK), downstream of Horsham sewage treatment works (51°03′19″N; 0°22′02″W) on 21 April 2008. We sorted the males by size (by measuring to the nearest centimeter) before introducing them to the spawning tanks, so that each tank contained a restricted size range of male fish. Thirteen breeding tanks were used, with a total of 117 fish, including 75 males (Table 1).

### Sampling of fish and morphometric analyses

We removed adult fish from the breeding tanks within 24 hr of spawning (5 days after collection from the wild), anesthetized them with 1:10,000 benzocaine, and measured the following end points for each adult male/intersex fish: *a*) length; *b*) weight; *c*) age (using counts of scale annuli); *d*) gonad histology; *e*) roughness of skin [on a scale of 0 (smooth) to 3 (large, visible tubercles all over skin of fish)]; *f*) sperm density; and *g*) sperm viability (assessed using trypan blue exclusion) (Hackett and MacPherson 1965). In addition, we collected fin clips from all adult fish and preserved them in 100% ethanol for genetic profiling and parentage analysis.

### Sampling of fry

The fry started to hatch approximately 1 week after spawning. We randomly sampled fry from the tanks 3–4 days posthatch and terminally anesthetized them before preserving them in absolute ethanol for DNA microsatellite analyses.

### Quantification of intersex index

We assessed gonadal histology using standard histological techniques as described by Jobling et al. (2006). We assigned each male fish a numerical score (intersex index) to classify the level of gonadal disruption based on the number of oocytes present in the testes (Jobling et al. 2006). Each of six sections was scored separately, and a mean score was calculated for each fish. Here we define intersex severity using intersex index values as follows: 0, nonintersex (normal male); > 0 but < 2, mildly intersex; ≥ 2 but < 4, moderately intersex; ≥ 4, severely intersex. Collectively, we refer to both male and intersex fish as “males.” The presence of female-like reproductive ducts (ovarian cavities) (Nolan et al. 2001) was also recorded but not included in the intersex index. Ovarian cavities in males can occur as a consequence of exposure to estrogen during early life. Oocytes in the testis, however, is a progressive condition, increasing with the level of EDC exposure and age of the fish. Our approach is consistent with that previously described for intersex indices in roach (Jobling et al. 2006).

### Microsatellite genotyping

We extracted DNA from the fin tissues of the adult fish, as well as from the fry, using the Chelex protocol (Estoup et al. 1996). We then genotyped all adult fish and selected fry from each tank using seven variable microsatellite loci (Hamilton and Tyler 2008). We genotyped each adult using an additional five microsatellites (CypG3, Lid1, Lid8, Lid11, Rru4) for individual genetic diversity calculations. We also genotyped four fry using these extra five genotypes, when the seven were unable to resolve parentage. We used the PROBMAX program (version 1.3; Danzmann 1997) for parentage analysis, except for six putative triploid fry from study 2 that inherited both maternal alleles and were assigned manually. Parentage analyses were based on samples of 52–58 fry from each tank in the 2006 study and 104 from each tank in the 2008 study.

A single triploid adult male was identified in study 2. This fish did not reproduce, and no milt was obtained from it. Because triploid fish are sterile, this individual was excluded from analyses of reproductive success.

### Individual genetic diversity

We calculated two indices of individual genetic diversity (measures of how “inbred” each individual is): homozygosity by loci (HL) (Aparicio et al. 2006) and mean standardized *d*^2^ (Amos et al. 2001; Coulson et al. 1998), from the parental microsatellite genotypes using the IRmacroN3 (Amos 2010). HL is calculated using allele frequencies, whereas *d*^2^ is calculated using size differences of alleles for each microsatellite locus within an individual.

### Statistical analysis

To deduce the influence of intersex on reproductive performance of male fish, we fitted linear mixed-effect (LME) models using the proportion of offspring sired per male as the response variable. To control for differences between tanks, we added a random tank effect to each model to capture the assumption that the reproductive performance of each fish depends on the other fish in the same tank. A weighted LME model was used to correct for the heteroskedasticity observed in the data (i.e., the greater variance in reproductive success observed at low values of intersex index), and we used an exponential variance function structure for the weights (Pinheiro and Bates 2000). We included all explanatory variables known to influence, or suspected of influencing, reproductive performance in the maximal model. These were intersex index, presence of ovarian cavities, length, HL, *d*^2^, roughness, sperm viability, and sperm density. We ran analyses both including and excluding fish for which sperm density and/or sperm viability data were unavailable (one fish from study 1 and six from study 2). Full models showing the results of the analysis including and excluding sperm parameters are presented in Supplemental Material, Tables 1 and 2, respectively (doi:10.1289/ehp.1002555). For both studies, weight and age were highly correlated with length (correlation coefficients ≥ 0.80), so these were excluded from the analyses. Thus, the full model used in the analysis was as follows:


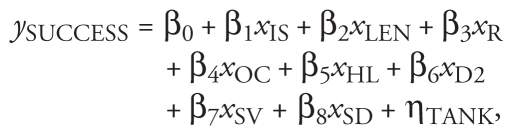


where IS is intersex index, LEN is length, R is roughness, OC is presence of ovarian cavities, D2 is *d*^2^, SV is sperm viability, and SD is sperm density. The parameters β_i_, for *i* = 1, . . . 8, and the tank effects were estimated using the LME function in R (version 2.8.1) (Ihaka and Gentlemen 1996). The likelihood ratio test (LRT) *p*-value for comparing the model with and without tank effects was > 0.99, suggesting that no tank effects are present in the data. To identify the key factors associated with reproductive performance, we obtained minimal adequate models using the stepwise backward procedure, until all selected variables had *p*-values < 0.1 calculated using an LRT. *p*-Values presented here were derived using an LRT based on model simplification of maximum-likelihood versions of the mixed models. In addition, we used permutation tests to verify the significance of terms with LRT *p*-values < 0.05 in the minimal adequate mixed-effects models (Table 1). Empirical distributions of the slope of the relationship between proportion paternity and each of the fixed effects in our minimal adequate models were created by shuffling randomly the paternities of fish within tanks, while maintaining the tank structure of the trials. *p*-Values for the permutation tests were calculated as the proportion of permutation slopes, based on 1,000 permutations, that were at least as extreme as the observed slope in the minimal adequate model.

## Results and Discussion

### Gonadal disruption in males

**Figure 1:**
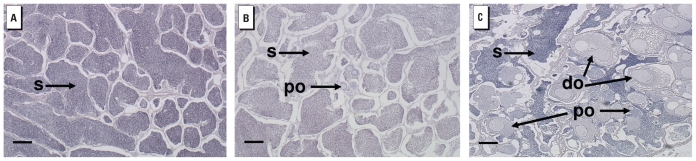
Histological sections from gonads of male fish showing different degrees of intersexuality. (*A*) Nonintersex male fish (intersex index = 0); the lobules of the testis are full of mature spermatozoa (s). (*B*) Mildly intersex fish (intersex index = 0.33); small numbers of primary oocytes (po) were found among tissue that consisted mainly of mature spermatozoa. (*C*) Severely intersex fish (intersex index = 4.8); the gonad consisted of large numbers of primary oocytes, as well as some oocytes in more advanced stages and some that were degenerating (do) and/or vacuolated, all set among male tissue, most of which (in this fish) consisted of mature spermatozoa. Bars = 100 μm.

We found evidence of feminization of male fish in both the rivers Bourne (study 1) and Arun (study 2), although the prevalence and severity of the condition differed between studies [see Supplemental Material, Figure 1 (doi:10.1289/ehp.1002555)]. Two of the 38 males (5.2%) from the River Bourne had ovarian cavities, and 15 (39%) had oocytes in their testes. Most of these intersex fish (13 of 15) were mildly feminized (had a low intersex index, with few oocytes in their testes). Only 2 were moderately affected, and we observed no severely intersex fish (high intersex index) in this study. In contrast, almost all of the 76 males from the River Arun used in study 2 had disrupted gonads: 88% had ovarian cavities, and 41% had oocytes in their testes. Of the intersex males used in this study, 18 were mildly disrupted, 9 were moderately intersex, and 4 were severely intersex. Examples of gonadal sections from males with different intersex indices are shown in Figure 1.

### Male reproductive success

In both studies, we observed considerable variations in male reproductive success. For example, in two tanks from study 1 and three tanks from study 2, a single male (out of either five or six present) sired > 50% of the offspring. Nevertheless, in both studies, most males (95% in study 1 and 91% in study 2), including intersex fish of all severities, sired offspring, demonstrating that in the scenarios represented by these experiments, most intersex fish were able to participate in spawning [see Supplemental Material, Figure 2 (doi:10.1289/ehp.1002555)]. Several factors significantly influenced male reproductive success (Table 1).

### Study 1: factors influencing reproduction in a mildly intersex population

**Figure 2:**
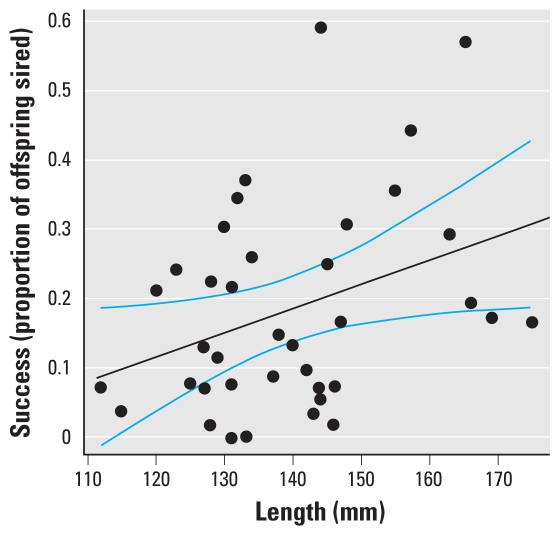
The relationship between length and reproductive success in study 1 for all 38 male fish. The black line indicates the line of best fit, and the blue lines indicate the 95% confidence limits.

**Figure 3:**
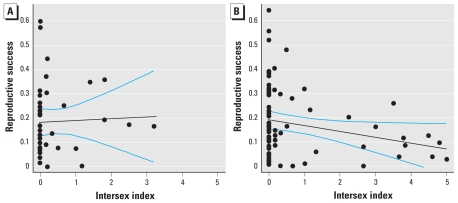
The relationship between severity of intersex and reproductive success of all “male” fish in (*A*) study 1 and (*B*) study 2. In cases in which more than one fish had the same values, data points overlie each other; thus, individual data points are not always visible (this is particularly the case among the less severely intersex fish). The black lines indicate the lines of best fit, and the blue lines indicate the 95% confidence limits.

In study 1, males of greater length were significantly more successful at siring offspring (LME model coefficient = 0.0036, *p* = 0.036; Table 1, Figure 2). Other studies have also found larger male fish to be more successful in competitive breeding scenarios (Fessehaye et al. 2006; Jacob et al. 2009), and across various taxa, dominance is related to body size (Qvarnström and Forsgren 1998). Roach have a “lek-like” breeding system (Wedekind 1996) in which a number of males occupy the spawning site, and females enter the area specifically to spawn with the competing males. Larger males of the European minnow (*Phoxinus phoxinus*), which also has a lek-like spawning strategy, are better able to defend spawning territory and have greater reproductive success (Jacob et al. 2009); this may have been the case in our study. We observed that neither intersex index nor the presence of ovarian cavities was significantly correlated with male reproductive performance in study 1 [*p*-values from LME models were 0.36 and 0.17, respectively; see Figure 3A and Supplemental Material, Table 1 (doi:10.1289/ehp.1002555)].

### Study 2: factors influencing reproduction in a population containing severely intersex males

We found a significant negative relationship between intersex index and reproductive success in study 2 (LME model coefficient = −0.029, *p* < 0.0001; Table 1, Figure 3B); more severely feminized fish had reduced success. Within the limits of our model (between intersex indices 0 and 5), the intersex condition decreased the proportion of offspring sired by affected individuals in each tank by 2.9% per unit increase in intersex index, when all other variables in the model are held constant (Table 1). Thus, the intersex condition reduced the average contribution to the offspring within each tank from 19% for nonintersex males (intercept for model with success and intersex only) to 4.5% for fish with an intersex index of 5. This represents a relative decrease in reproductive performance of 76%, or 15% per intersex unit, assuming a linear relationship (as shown in Figure 3B). These slopes should not be used to extrapolate beyond the group sizes used in these trials, but they nonetheless demonstrate a significant adverse effect of intersex on reproductive performance.

The difference in the consequence of intersexuality on reproductive success between studies 1 and 2 is likely due to the increased range of intersex indices observed in the fish used in study 2: Study 1 only included 2 fish that were moderately intersex, whereas study 2 included 13 fish with an intersex index > 2. This fact, together with the increase in the total number of males used in the second study, afforded a wider and more powerful analysis of the relationship between intersexuality and reproductive success. Moreover, males in the second study were size sorted (reducing the mean maximum size difference within each tank from 40 mm in study 1 to 12 mm in study 2) in order to reduce the potential confounding effect of size on reproductive performance. Indeed, the small size differences that were still present in the second study did not significantly affect the reproductive hierarchy (*p* = 0.076; Table 1). We did, however, observe a statistically significant relationship between the presence of ovarian cavities and reproductive success (LME model coefficient = 0.045, *p* = 0.05; Table 1), albeit the trend was opposite to that expected, with males with ovarian cavities performing better than those without.

The observed reduction in reproductive success of intersex fish could have resulted from a combination of factors, including a reduced ability to release milt due to blocked or obstructed sperm ducts, reduced sperm quality, reduced hatching success or survival of fry, and/or reduced ability to compete with other males or attract females. Using *in vitro* techniques, Jobling et al. (2002) found that intersex fish have reduced sperm quality. This may have been the case in our study, because we found that sperm viability was significantly correlated with reproductive success [LME model coefficient = 0.0023, *p* = 0.001; see Supplemental Material, Table 3 (doi:10.1289/ehp.1002555)].

We also demonstrated a significant association between the internal genetic diversity measure HL and reproductive success in study 2. However, the trend was opposite to that expected, with the more “inbred” fish being more successful (LME model coefficient = 0.14, *p* = 0.019; Table 1). “Outbred” individuals of several vertebrate species have been shown to have greater reproductive success (Amos et al. 2001); however, the influence of inbreeding on reproductive success of fish is less clear. Although experimentally generated inbred male and female tilapia (*Oreochromis niloticus*) have been found to have reduced reproductive success (Fessehaye et al. 2009), another study found no effects of parental internal genetic diversity on the reproductive output of Atlantic salmon (*Salmo salar*) (Garant et al. 2005).

### Analysis of the combined results from both studies

A statistically significant negative relationship between severity of intersex and reproductive success was also apparent in the analysis of the combined data from the two experiments (LME model coefficient = −0.029, *p* = 0.0001; Table 1). Across all tanks, the percentage of intersex fish not reproducing was higher (13%) than the percentage of nonintersex males not reproducing (4.4%), although this apparent consequence of intersexuality was not statistically significant at the 95% level (Fisher’s exact test, *p* = 0.15).

In contrast to the analysis of study 2 data alone, we found no significant association between the presence of ovarian cavities and reproductive success when the data were combined [*p* = 0.35; see Supplemental Material, Table 1 (doi:10.1289/ehp.1002555)]; indeed, the full model that included sperm quality measures demonstrated a significant negative relationship between the presence of ovarian cavities and reproductive success when we combined the data from the two studies [LME model coefficient = −0.030, *p* = 0.041; see Supplemental Material, Table 2 (doi:10.1289/ehp.1002555)] in contrast to the positive relationship revealed in study 2. Hence, the relationship observed between these factors was inconsistent and the implications of this association remain unclear.

In addition, after analysis of the combined data, we observed that fish with more prominent secondary sexual characteristics (breeding tubercles causing roughness of the body surface) were more successful in fathering offspring (LME model coefficient = 0.031, *p* = 0.022; Table 1). Although it is unclear whether there is any female choice in roach, it has been suggested that breeding tubercles are used by females as a cue for choosing a high-quality mate, and males with large breeding tubercles exhibit significantly more active courtship behavior (Kortet et al. 2004). The presence of tubercles has also been found to be important in the reproductive success of minnows (Jacob et al. 2009).

Other significant trends observed after analysis of the separate data sets (length and sperm viability in studies 1 and 2, respectively) were also reflected in analyses of the combined data; reproductive success was significantly positively correlated both with body length (*p* = 0.035; Table 1) and sperm viability [*p* = 0.0007; see Supplemental Material, Table 3 (doi:10.1289/ehp.1002555)].

### Implications and limitations

Our results, although demonstrating that intersex fish are able to reproduce in a realistic competitive breeding scenario, nonetheless indicate a significant reduction in reproductive capability of severely intersex fish, as well as an effect on parentage outcome in the population as was suggested (but not proved) by our previous work using *in vitro* fertilization techniques (Jobling et al. 2002). The implications of the reduced reproductive capacity of a proportion of fish in a given population are largely unknown but could potentially include diminished recruitment. Likewise, effective population sizes, which are important for long-term maintenance of genetic variability, could be affected, especially in combination with other documented effects of laboratory-based EDC exposure, such as sex reversal, reduced female fecundity, and alterations in timing of reproduction. Finally, if susceptibility to EDC exposure has a genetic basis, a skew in success toward less feminized individuals provides a mechanism for the evolution of tolerance to the feminizing effects of EDCs.

It is difficult to extrapolate the data presented here directly to the wild because too much is unknown about the actual spawning scenario of wild roach. For instance, as far as we are aware, the size of breeding colonies in the wild is unknown and may vary widely. Likewise, the sex ratio at spawning sites is largely unknown but potentially could be more male biased in the wild than the 2:1 male:female ratio used here, because males congregate at spawning sites, whereas females enter only to spawn (Diamond 1985; Wedekind 1996).

## Conclusion

We conclude that if severely intersex fish are present in a river, there could be implications for the fish population concerned, whereas if only mildly intersex fish are present, it seems likely that the effects (if there are any) will be less severe. This conclusion needs to be considered in the context of available data concerning the prevalence of intersex fish in rivers. For instance, in U.K. rivers the proportion of male roach that are moderately to severely intersex (intersex index ≥ 2) is generally < 10%, and the proportion of severely intersex fish alone (intersex index ≥ 4) is < 4%, even in highly contaminated sites, based on the combined data from surveys undertaken previously (Jobling et al. 1998, 2002, 2006). However, data specifying the severity of gonadal disruption in individual fish (as opposed to the overall proportion of intersex fish in a river) are rare; those studies that have provided such detail suggest a low proportion of severely intersex fish in river systems in Europe and the United States (Bjerregaard et al. 2006; Blazer et al. 2007; Jobling et al 1998; Minier et al. 2000). Locally high prevalences of intersex have been reported by Hinck et al. (2009) for other fish species in the United States (91% for largemouth bass and 73% for smallmouth bass), although the authors did not describe the severity of intersex observed in individual fish. Therefore, currently available information would suggest that, because the majority of feminized males at effluent-impacted sites are mildly intersex, it seems unlikely that the intersex condition alone would result in short-term population crashes on the scale of those observed in previous long-term (albeit high concentration) EDC exposure studies (e.g., Kidd et al. 2007). It is nevertheless clear that further knowledge of roach populations and the application of population modeling to these collective data would be helpful in determining the long-term consequences of the intersex condition.

## Figures and Tables

**Table 1:** Variables associated with reproductive success of male roach (*Rutilus rutilus*) obtained by LME models in the two breeding studies.

Study	No. of males	No. of tanks	Variable	Coefficient	LRT *p*-value	Permutation *p*-value
1	38	7	Length	0.0036	0.036	0.021

2	75	13	Intersex	−0.029	< 0.0001	0.001
			Ovarian cavities	0.045	0.05	0.0001
			Length	0.00062	0.076	NA
			Genetic diversity (HL)	0.14	0.019	0.0001

Combined data set	113	20	Intersex	−0.029	0.0001	0.015
			Length	0.00080	0.035	0.027
			Roughness	0.031	0.022	0.048

Values are those retained after stepwise elimination of variables with *p*-values > 0.1 calculated using LRTs; hence, not all of the variables analyzed are shown. Full models are shown in Supplemental Material (doi:10.1289/ehp.1002555); Supplemental Material, Table 1 shows results of full models excluding sperm parameters, and Supplemental Material, Tables 2 and 3 show results of models including sperm density and viability but excluding some individual fish for which sperm data were not available. NA, not applicable; permutation tests were carried out only on variables giving LRT *p*-values < 0.05.

## References

[b1-ehp-119-306] Allen Y, Scott AP, Matthiessen P, Haworth S, Thain JE, Feist S (1999). Survey of estrogenic activity in United Kingdom estuarine and coastal waters and its effects on gonadal development of the flounder *Platichthys flesus*. Environ Toxicol Chem.

[b2-ehp-119-306] Amos W (2010). Computer Programs: IRmacroN3.

[b3-ehp-119-306] Amos W, Wilmer JW, Fullard K, Burg TM, Croxall JP, Bloch D (2001). The influence of parental relatedness on reproductive success. Proc R Soc Lond B.

[b4-ehp-119-306] An W, Hu J, Giesy JP, Yang M (2009). Extinction risk of exploited wild roach (*Rutilus rutilus*) populations due to chemical feminization. Environ Sci Technol.

[b5-ehp-119-306] Animals (Scientific Procedures) Act (1986). http://tna.europarchive.org/20100413151426/http://www.archive.official-documents.co.uk/document/hoc/321/321-xa.htm.

[b6-ehp-119-306] Aparicio JM, Ortego J, Cordero PJ (2006). What should we weigh to estimate heterozygosity, alleles or loci?. Mol Ecol.

[b7-ehp-119-306] Bjerregaard LB, Korsgaard B, Bjerregaard P (2006). Intersex in wild roach (*Rutilus rutilus*) from Danish sewage effluent-receiving streams. Ecotoxicol Environ Safe.

[b8-ehp-119-306] Blazer VS, Iwanowicz LR, Iwanowicz DD, Smith DR, Young JA, Hedrick JD (2007). Intersex (testicular oocytes) in smallmouth bass from the Potomac River and selected nearby drainages. J Aquat Anim Health.

[b9-ehp-119-306] Coulson TN, Pemberton JM, Albon SD, Beaumont M, Marshall TC, Slate J (1998). Microsatellites reveal heterosis in red deer. Proc Biol Sci.

[b10-ehp-119-306] Danzmann RG (1997). PROBMAX: a computer program for assigning unknown parentage in pedigree analysis from known genotypic pools of parents and progeny. J Hered.

[b11-ehp-119-306] De Metrio G, Corriero A, Desantis S, Zubani D, Cirillo F, Deflorio M (2003). Evidence of a high percentage of intersex in the Mediterranean swordfish (*Xiphias gladius* L.). Mar Pollut Bull.

[b12-ehp-119-306] Desbrow C, Routledge EJ, Brighty GC, Sumpter JP, Waldock M (1998). Identification of estrogenic chemicals in STW effluent. 1. Chemical fractionation and in vitro biological screening. Environ Sci Technol.

[b13-ehp-119-306] Diamond M (1985). Some observations of spawning by roach, *Rutilus rutilus* L., and bream, *Abramis brama* L., and their implications for management. Aquacult Res.

[b14-ehp-119-306] Estoup A, Largiader CR, Perrot E, Chourrout D (1996). Rapid one-tube DNA extraction for reliable PCR detection of fish polymorphic markers and transgenes. Mol Mar Biol Biotech.

[b15-ehp-119-306] Fessehaye Y, Bovenhuis H, Rezk MA, Crooijmans R, van Arendonk JAM, Komen H (2009). Effects of relatedness and inbreeding on reproductive success of Nile tilapia (*Oreochromis niloticus*). Aquaculture.

[b16-ehp-119-306] Fessehaye Y, El-bialy Z, Rezk MA, Crooijmans R, Bovenhuis H, Komen H (2006). Mating systems and male reproductive success in Nile tilapia (*Oreochromis niloticus*) in breeding hapas: a microsatellite analysis. Aquaculture.

[b17-ehp-119-306] Garant D, Dodson JD, Bernatchez L (2005). Offspring genetic diversity increases fitness of female Atlantic salmon (*Salmo salar*). Behav Ecol Sociobiol.

[b18-ehp-119-306] Grist EPM, Wells NC, Whitehouse P, Brighty G, Crane M (2003). Estimating the effects of 17 alpha-ethinylestradiol on populations of the fathead minnow *Pimephales promelas*: are conventional toxicological endpoints adequate?. Environ Sci Technol.

[b19-ehp-119-306] Gutjahr-Gobell RE, Zaroogian GE, Horowitz DJB, Gleason TR, Mills LJ (2006). Individual effects of estrogens on a marine fish, Cunner (*Tautogolabrus adspersus*), extrapolated to the population level. Ecotoxicol Environ Safe.

[b20-ehp-119-306] Hackett AJ, MacPherson JW (1965). A method for differential staining of bovine spermatozoa after extension in sterile milk. Can Vet J.

[b21-ehp-119-306] Hahlbeck E, Griffiths R, Bengtsson BE (2004). The juvenile three-spined stickleback (*Gasterosteus aculeatus* L.) as a model organism for endocrine disruption—I. Sexual differentiation. Aquat Toxicol.

[b22-ehp-119-306] Hamilton PB, Tyler CR (2008). Identification of microsatellite loci for parentage analysis in roach *Rutilus rutilus* and 8 other cyprinid fish by cross-species amplification, and a novel test for detecting hybrids between roach and other cyprinids. Mol Ecol Resour.

[b23-ehp-119-306] Hinck JE, Blazer VS, Schmitt CJ, Papoulias DM, Tillitt DE (2009). Widespread occurrence of intersex in black basses (*Micropterus* spp.) from US rivers, 1995–2004. Aquat Toxicol.

[b24-ehp-119-306] Hotchkiss AK, Rider CV, Blystone CR, Wilson VS, Hartig PC, Ankley GT (2008). Fifteen years after “Wingspread”—environmental endocrine disrupters and human and wildlife health: where we are today and where we need to go. Toxicol Sci.

[b25-ehp-119-306] Ihaka R, Gentlemen R (1996). R: a language for data analysis and graphics. J Comput Graph Stat.

[b26-ehp-119-306] Jacob A, Evanno G, Renai E, Sermier R, Wedekind C (2009). Male body size and breeding tubercles are both linked to intrasexual dominance and reproductive success in the minnow. Anim Behav.

[b27-ehp-119-306] Jobling S, Coey S, Whitmore JG, Kime DE, Van Look KJW, McAllister BG (2002). Wild intersex roach (*Rutilus rutilus*) have reduced fertility. Biol Reprod.

[b28-ehp-119-306] Jobling S, Nolan M, Tyler CR, Brighty G, Sumpter JP (1998). Widespread sexual disruption in wild fish. Environ Sci Technol.

[b29-ehp-119-306] Jobling S, Williams R, Johnson A, Taylor A, Gross-Sorokin M, Nolan M (2006). Predicted exposures to steroid estrogens in UK rivers correlate with widespread sexual disruption in wild fish populations. Environ Health Perspect.

[b30-ehp-119-306] Kidd KA, Blanchfield PJ, Mills KH, Palace VP, Evans RE, Lazorchak JM (2007). Collapse of a fish population after exposure to a synthetic estrogen. Proc Natl Acad Sci USA.

[b31-ehp-119-306] Kortet R, Taskinen J, Vainikka A, Ylonen H (2004). Breeding tubercles, papillomatosis and dominance behaviour of male roach (*Rutilus rutilus*) during the spawning period. Ethology.

[b32-ehp-119-306] Kortet R, Vainikka A, Rantala MJ, Jokinen I, Taskinen J (2003). Sexual ornamentation, androgens and papillomatosis in male roach (*Rutilus rutilus*). Evol Ecol Res.

[b33-ehp-119-306] Lange A, Katsu Y, Ichikawa R, Paull GC, Chidgey LL, Coe TS (2008). Altered sexual development in roach (*Rutilus rutilus*) exposed to environmental concentrations of the pharmaceutical 17α-ethinylestradiol and associated expression dynamics of aromatases and estrogen receptors. Toxicol Sci.

[b34-ehp-119-306] Länge R, Hutchinson TH, Croudace CP, Siegmund F, Schweinfurth H, Hampe P (2001). Effects of the synthetic estrogen 17α-ethinylestradiol on the life-cycle of the fathead minnow (*Pimephales promelas*). Environ Toxicol Chem.

[b35-ehp-119-306] Miller DH, Ankley GT (2004). Modeling impacts on populations: fathead minnow (*Pimephales promelas*) exposure to the endocrine disruptor 17β-trenbolone as a case study. Ecotoxicol Environ Safe.

[b36-ehp-119-306] Minier C, Caltot G, Leboulanger F, Hill EM (2000). An investigation of the incidence of intersex fish in Seine-Maritime and Sussex regions. Analusis.

[b37-ehp-119-306] Nolan M, Jobling S, Brighty G, Sumpter JP, Tyler CR (2001). A histological description of intersexuality in the roach. J Fish Biol.

[b38-ehp-119-306] Palace VP, Wautier KG, Evans RE, Blanchfield PJ, Mills KH, Chalanchuk SM (2006). Biochemical and histopathological effects in pearl dace (*Margariscus margarita*) chronically exposed to a synthetic estrogen in a whole lake experiment. Environ Toxicol Chem.

[b39-ehp-119-306] Penaz M, Svobodova Z, Barus V, Prokes M, Drastichova J (2005). Endocrine disruption in a barbell, *Barbus barbus* population from the River Jihlava, Czech Republic. J Appl Ichthyol.

[b40-ehp-119-306] Pinheiro JC, Bates DM (2000). Mixed-Effects Models in S and S-PLUS.

[b41-ehp-119-306] Purdom CE, Hardiman PA, Bye VVJ, Eno NC, Tyler CR, Sumpter JP (1994). Estrogenic effects of effluents from sewage treatment works. Chem Ecol.

[b42-ehp-119-306] Qvarnström A, Forsgren E (1998). Should females prefer dominant males?. Trends Ecol Evol.

[b43-ehp-119-306] Stentiford GD, Feist SW (2005). First reported cases of intersex (ovotestis) in the flatfish species dab *Limanda limanda*: Dogger Bank, North Sea. Mar Ecol Prog Ser.

[b44-ehp-119-306] Sumpter JP, Johnson AC (2008). 10th Anniversary perspective: reflections on endocrine disruption in the aquatic environment: from known knowns to unknown unknowns (and many things in between). J Environ Monit.

[b45-ehp-119-306] Sun Q, Deng S, Huang J, Shen G, Yu G (2008). Contributors to estrogenic activity in wastewater from a large wastewater treatment plant in Beijing, China. Environ Toxicol Pharmacol.

[b46-ehp-119-306] Wedekind C (1996). Lek-like spawning behaviour and different female mate preferences in roach (*Rutilus rutilus*). Behaviour.

[b47-ehp-119-306] Werner J, Palace VP, Wautier KG, Mills KH, Chalanchuk SM, Kidd KA (2006). Reproductive fitness of lake trout (*Salvelinus namaycush*) exposed to environmentally relevant concentrations of the potent estrogen ethynylestradiol (EE2) in a whole lake exposure experiment. Sci Mar.

